# Dynamical Sampling with Langevin Normalization Flows

**DOI:** 10.3390/e21111096

**Published:** 2019-11-10

**Authors:** Minghao Gu, Shiliang Sun, Yan Liu

**Affiliations:** 1 School of Computer Science and Technology, East China Normal University, 3663 North Zhongshan Road, Shanghai 200241, China; guminghao1081@gmail.com; 2 Shanghai Institute of Intelligent Science and Technology, Tongji University, Shanghai 201804, China; 3 School of Data Science and Engineering, East China Normal University, 3663 North Zhongshan Road, Shanghai 200241, China; yliu@cc.ecnu.edu.cn

**Keywords:** normalization flows, Langevin diffusions, Langevin normalization flows, Monte Carlo sampling

## Abstract

In Bayesian machine learning, sampling methods provide the asymptotically unbiased estimation for the inference of the complex probability distributions, where Markov chain Monte Carlo (MCMC) is one of the most popular sampling methods. However, MCMC can lead to high autocorrelation of samples or poor performances in some complex distributions. In this paper, we introduce Langevin diffusions to normalization flows to construct a brand-new dynamical sampling method. We propose the modified Kullback-Leibler divergence as the loss function to train the sampler, which ensures that the samples generated from the proposed method can converge to the target distribution. Since the gradient function of the target distribution is used during the process of calculating the modified Kullback-Leibler, which makes the integral of the modified Kullback-Leibler intractable. We utilize the Monte Carlo estimator to approximate this integral. We also discuss the situation when the target distribution is unnormalized. We illustrate the properties and performances of the proposed method on varieties of complex distributions and real datasets. The experiments indicate that the proposed method not only takes the advantage of the flexibility of neural networks but also utilizes the property of rapid convergence to the target distribution of the dynamics system and demonstrate superior performances competing with dynamics based MCMC samplers.

## 1. Introduction

In machine learning, Bayesian inference [1] and Bayesian optimization [2], complex probabilistic models typically require the calculation of intractable and high-dimension integrals. For example, for a classification task, we need to predict the class of instances. We assume that $p\left(y_{*}|x_{*},D\right)=\int p\left(y_{*}|x_{*},\theta \right)p\left(\theta |D\right)d\theta$ is the prediction model, where $x_{*}$ represents the instance, $y_{*}$ represents the class, D represents the data, $p(y_{*}|x_{*},\theta )$ is the likelihood function and p(θ|D) is the posterior distribution. When the probabilistic model becomes complex, this integral is intractable. Generally, two kinds of methods are used to approximate the integral, which are Markov chain Monte Carlo (MCMC) [3,4] and variational inference (VI) [5,6]. MCMC is a powerful framework, which is widely used to deal with the complex and intractable probabilistic models [7,8,9]. MCMC methods approximate the complex probability distributions by a large number of samples which are sampled from a Markov chain iteratively. They serve as a fundamental approach in probabilistic inference, which provides the asymptotically unbiased estimation for the probabilistic models, while VI gives the deterministic approximation for the target distributions [10].

Recent MCMC methods can be divided into two aspects. One class of the MCMC methods is slice sampling [11] and the other one is dynamical sampling [12,13]. The main problem of the slice sampler is that when sampling from the distributions with high dimensions, solving the slice interval can be very difficult. Utilizing the dynamics system to construct an efficient Markov chain is commonly employed [14,15,16]. Hamiltonian Monte Carlo (HMC) [14] is one of the dynamics based methods, which has multiple attractive properties concerning rapid explorations of the state space and high acceptance rate of the samples. HMC exploits Hamiltonian dynamics to construct efficient Markov chain Monte Carlo, which has become increasingly popular in machine learning and statistics. Since HMC uses the gradient information of the target distribution, it can explore the state space much more efficiently than the random-walk proposals [17], which ensures the rapid convergence of the sampler. Since it has the property of volume conservation, HMC is able to propose large moves with a higher acceptance rate.

HMC and its further developments [18,19,20,21,22,23] exploit the gradient information of the target distribution to explore the state space. Nevertheless, since the step size of the leapfrog is difficult to choose, there exists the correlation between neighbor samples and thus the high autocorrelation may occur. Though we can enlarge the step size of the leapfrog, it will waste a lot of computation resources. Moreover, they tend to fail when the target distributions are multi-modal [21,24,25,26]. These MCMC methods usually fail to move from one mode to another because such a move requires passing through low probability regions. These places have large boundary gradients which prevent samplers from traveling through the modes. Therefore, designing an effective sampler for multi-modal distributions has remained a significant challenge.

The disadvantages of the current methods motivate us to design a powerful sampler which can have not only low autocorrelation but also accurate estimation for the target distribution. In this paper, a new sampling method called Langevin normalization flows Monte Carlo (NFLMC) is proposed. We introduce Langevin diffusions to the normalization flows (NFs) [27] to construct a new sampler. The main idea of this method is to train a variational distribution to approximate the target distribution, whose parameters are determined by the neural networks. With the idea of Langevin diffusions, we design new transformation functions for NFs which have the properties of rapid convergence to the target distribution and better approximation to the target distributions. Since we exploit the gradient information of the target distributions, the calculation of the integrals of the Kullback-Leibler (KL) divergence is intractable. So we use the Monte Carlo estimator to calculate the KL divergence. However, the KL divergence calculated by Monte Carlo estimator may be negative in the process of training, which would mislead the final results, so we propose a new loss function to train the NFLMC sampler.

The main contributions of this paper can be summarized as follows. (1) We introduce Langevin diffusions to normalization flows to construct a novel Monte Carlo sampler. (2) We propose the modified KL divergence as the loss function to train the sampler, which ensures that the proposed method can converge to the target distribution. (3) The proposed method achieves better performances in multi-modal sampling and varieties of complex distributions. (4) we do not need the Metropolis-Hasting procedure [28] to adjust the sampler compared with MCMC samplers. (5) A number of experiments verify the theoretical results and practical value. We apply the proposed method to varieties of distributions and supervised classification tasks using Bayesian logistic regression. The proposed method is compared with state-of-the-art dynamics based MCMC methods [24,29,30] in the autocorrelation rate and convergence speed. The experiments demonstrate that the NFLMC method has a superior performance in sampling complex posterior distributions.

The rest of this article is organized as follows. In Section 2, we review the preliminary of our study, including the introduction of variational inference with normalization flows and Langevin diffusions. In Section 3, we introduce our Langevin normalization flows and describe the transformation functions. In Section 4, we propose the Langevin normalization flows Monte Carlo sampler. Experiments and analysis are given in Section 5. In Section 6, we conclude this paper and discuss the future work.

## 2. Preliminary

### 2.1. Normalization Flows

The normalization flows [27] were first introduced to deal with the flexible and complex posterior distributions in the context of variational inference. It is a powerful approach to generate arbitrary posterior distributions utilizing a sequence of invertible transformation. In other words, the initial density will transform to a valid probability distribution through iteratively applying the normalization flows. Given the observed data x, the normalization flows start with an initial variable $z_{0}$ generated from a simple distribution *q*, which has the analytical probability density and then repeatedly apply an invertible transformation function $f_{\theta }$ which is parameterized by θ. After a sequence of iterations, a complex and flexible distribution of $z_{T}$ will be obtained. It takes the form as follows:

(1) $$z_{0}∼q\left(z_{0}|x\right),z_{t}∼f_{\theta }\left(z_{t-1}|x\right),\forall t=1\ldots T.$$

Since the Jacobian determinant of each transformation $f_{\theta }$ can be calculated, we can obtain the final distribution $\pi_{u_{T}}$ through the following equation.

(2) $$\ln\left[\pi_{u_{T}}\left(z_{T}|x\right)\right]=\ln\left[q(z_{0}|x)\right]-\sum_{t=1}^{T}\ln\left(\det\left|\frac{\partial z_{t}}{\partial z_{t-1}}\right|\right).$$

**Figure 1:**
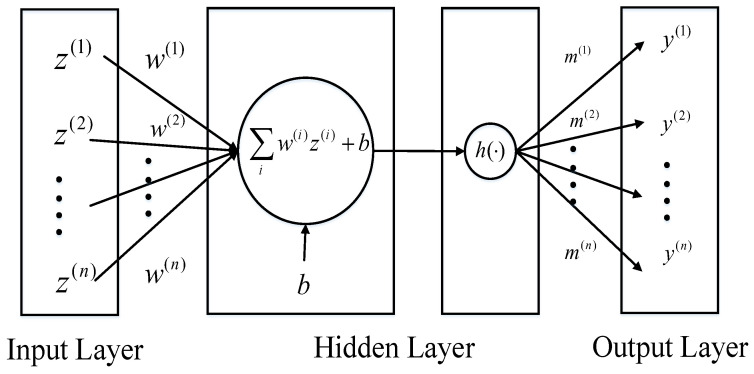
The multi-layer perceptron with one hidden layer and a single unit.

To make Equation (2) tractable, the Jacobian determinant of each transformation function $f_{\theta }$ should be carefully designed to satisfy two main properties. First, the transformation function $f_{\theta }$ is easy to invert. Second, the Jacobian determinant should be tractable. We assume that $z_{0}$ comes from a simple distribution $q(z_{0}|x)$ and $z_{T}=f_{\theta }\left(z_{0}\right)$. When calculating the probability of $z_{T}$ in Equation (2), we need to calculate the Jacobian determinant and use $f^{-1}\left(z_{T}\right)$ to calculate $z_{0}$. So the transformation function $f_{\theta }$ should be easy to invert and the Jacobian determinant should be tractable. Generally, the invertible transformation function $f_{\theta }$ with known Jacobian determinant [27] is defined as:

(3) $$f_{\theta }\left(z_{t-1}\right)=z_{t-1}+mh\left(w^{T}z_{t-1}+b\right),$$

where h(·) represents the nonlinear function, $m=[m^{1},m^{2},\ldots ,m^{n}]$ and $w=[w^{1},w^{2},\ldots ,w^{n}]$ are parameter vectors and *b* is the scalar and *n* is the dimension of the parameter vectors. So $mh(w^{T}z_{t-1}+b)$ can be viewed as a multi-layer perceptron with one hidden layer and a single unit, which is demonstrated in Figure 1.

Real-valued non-volume preserving (RNVP) [31] develops a new transformation function, which makes the model more flexible. The main idea of RNVP is that coupling layers are used to construct the normalization flows. Assume that x is the original variable. The coupling layers can be defined as:

(4) $$\begin{array}{cc} y_{1:d}\;\;\; & =x_{1:d}\\ y_{d+1:D} & =x_{d+1:D}⊙\exp\left(s\left(x_{1:d}\right)\right)+t\left(x_{1:d}\right), \end{array}$$

where function *s* represents the scale and *t* represents the translation. Both of them are neural networks. RNVP provides a more powerful and flexible posterior distribution for density estimation.

Recently, neural density estimators are widely used in the approximation of data distributions [32,33] and variational inference [27]. NFs and their further studies performs very well in modeling images, videos and audio [31,34,35,36,37].

### 2.2. Langevin Diffusions

Langevin dynamics is a common method to model molecular dynamics systems. A *D*-dimension Langevin diffusions are a time based stochastic process $x=(x_{t}),t\geq 0$ with stochastic sample paths, which can be defined as a solution to the stochastic differential equation taking the form as follows:

(5) $$dx_{t}=b\left(x_{t}\right)dt+\sigma \left(x_{t}\right)dW_{t},$$

where b(x) represents the drift vector, σ(x) represents the volatility matrix and $W=\left(W_{t}\right),t\geq 0$ represents a standard Wiener process [38]. Equation (5) gives the evolution of a random variable under Langevin diffusions but when it comes to the evolution of the probability density function, the diffusions should be described by Fokker-Planck equation [39]. We assume that u(x,t) represents the evolution of the probability density function, $x={\left[x_{1},x_{2},\ldots ,x_{D}\right]}^{T}$ and $V\left(x\right)=\sigma \left(x\right)\sigma {\left(x\right)}^{T}$. We set $b_{i}\left(x\right)$ to be the *i*-th term of the vector b(x) and $V_{ij}\left(x\right)$ to be the *i*-th row and the *j*-th column’s term of the matrix V(x). So the Fokker-Planck equation can be defined as follows:

(6) $$\begin{array}{cc} \frac{\partial }{\partial t}u\left(x,t\right)= & -\sum_{i=1}^{D}\frac{\partial }{\partial x_{i}}\left[b_{i}\left(x\right)u\left(x,t\right)\right]+\frac{1}{2}\sum_{i=1,j=1}^{D}\frac{\partial^{2}}{\partial x_{i}\partial x_{j}}\left[V_{ij}\left(x\right)u\left(x,t\right)\right]. \end{array}$$

If we have $u\left(x,t\right)=\pi_{g}\left(x\right),\forall t\in T$, then this process is stationary and $\pi_{g}$ can be viewed as the stationary distribution of the diffusion, which means that if $x_{t}∼\pi_{g}\left(x\right)$, then $x_{t+\epsilon }∼\pi_{g}\left(x\right)$, ∀ϵ>0 [40]. Langevin diffusion with stationary distribution $\pi_{g}$ can be defined by the stochastic differential equation [23]:

(7) $$dx_{t}=\frac{1}{2}\nabla \ln\pi_{g}\left(x_{t}\right)dt+dW_{t}.$$

The setting of b, σ and u(x,t) in Equation (7) makes $\frac{\partial u}{\partial t}=0$, which suggests that the invariant measure of Langevin diffusion is related to $\pi_{g}\left(x\right)$ [40].

Generally, solving the stochastic differential equations exactly is intractable. Since stochastic differential equations usually have strong coupling and nonlinearity, it is difficult to calculate the exact expression of its solution. So it is necessary to utilize the numerical discretization methods to approximate the solution to stochastic differential equations. Euler-Maruyama discretization [41] is one of the common approaches to obtain the approximate solution to the stochastic differential equation, which takes the form as:

(8) $$x_{t+1}=x_{t}-\frac{\epsilon^{2}}{2}\nabla_{x}\ln\pi_{g}\left(x_{t}\right)+\epsilon z_{t},$$

where $z_{t}∼N\left(z|0,I\right)$ and ϵ represents the step size.

It is noted that Langevin diffusions take advantage of the gradient information of the target distribution. The gradient information makes Langevin diffusions explore the state space efficiently. What’s more, Langevin diffusions contain the Wiener process that can be viewed as the random work. The random work helps to explore the state space extensively. The idea of Langevin diffusions are widely used in MCMC methods. Metropolis adjusted Langevin algorithm (MALA) [40] is one of the applications of Langevin diffusions. The main idea of MALA is to give the proposed state through Langevin diffusions, whose equation is given in Equation (8). MALA exploits the Metropolis-Hasting correction [28] to satisfy the detailed balance [42], which ensures that the samples generated from Langevin diffusions will converge to the target distribution. It is the gradient information of the target distribution that accelerates the convergence rate to the stationary distribution of MCMC. Although MALA do provide an efficient way for MCMC to sample from the target distribution, the autocorrelation among samples remains high.

Since NFs provide a more powerful and flexible posterior distribution for density estimation and MALA achieves rapid convergence to the target distribution, we maintain their advantages to develop a new sampler with appropriate training strategy, which can accurately sample from the target distribution with low autocorrelation.

## 3. Langevin Normalization Flows

### 3.1. Main Idea

Normalization flows [27,31] approximate the target distributions through a series of transformation functions. In order to approximate the target distributions efficiently and accurately, we utilize the information of the target distributions. Through exploiting the advantages of efficient exploration of Langevin diffusions, we propose a new normalization flow which is called Langevin normalization flows (NFL). We redesign the transformation functions through the gradient information of the target distribution, which helps us to approximate the target distributions precisely and efficiently.

Constructing the Langevin normalization flows has to satisfy two primary conditions. The first one is that the update of each step of the transformation function should be approximately invertible. The second one is that the determinant of the Jacobian and the inverse Jacobian of the transformation function must be tractable. In this way, we can ensure that the distribution obtained through the flows is able to converge to the target distribution.

We then describe the details of our proposed transformation functions for a single Langevin step. We assume that $x_{1:D}$ is the initial sample, where *D* is the number of the dimension of the sample. We first update a half of the sample. The transformation functions are as follows:

(9) $$\left\{\begin{array}{c} y_{1:d}\;\;=x_{1:d},\\ y_{d+1:D}=\left(x_{d+1:D}-\frac{\epsilon^{2}}{2}\nabla U{\left(x_{1:D}\right)}_{d+1:D}+\epsilon \cdot \exp\left(\sigma \left(x_{1:d}\right)\right)\right)\\ \;\;\;\;\;⊙\exp\left(S\left(x_{1:d}\right)\right)+T\left(x_{1:d}\right),\; \end{array}\right.$$

where σ(x) can be viewed as the Wiener process in Langevin diffusions. S(x) represents the logarithmic scale of the sample which is able to rescale the position of the sample. T(x) is the shift of the sample. σ(x), S(x) and T(x) are all controlled by the neural networks, where $W_{\sigma }$, $W_{S}$ and $W_{T}$ are their parameters. *U* is the energy function of the probability density function. In addition, ϵ represents the step size of the Langevin diffusions. It is noted that in Equation (9), we first utilize Langevin diffusions to generate samples and then we use neural networks to further adjust the samples. Since we only update $x_{d+1:D}$ and $y_{1:D}$ is the intermediate variable, $x_{1:d}$ should be updated then. It takes the form as:

(10) $$\left\{\begin{array}{c} z_{d+1:D}=y_{d+1:D},\\ z_{1:d}\;\;\;=\left(y_{1:d}-\frac{\epsilon^{2}}{2}\nabla U{\left(y_{1:D}\right)}_{1:d}+\epsilon \cdot \exp\left(\sigma \left(y_{d+1:D}\right)\right)\right)\\ \;\;\;\;\;⊙\exp\left(S\left(y_{d+1:D}\right)\right)+T\left(y_{d+1:D}\right), \end{array}\right.$$

where $z_{1:D}$ represents the final obtained state after applying the above transformation functions to $y_{1:D}$. The advantage of dividing x into two part is that Equation (9) generates $y_{d+1}$ and affects only $x_{d+1}$ while Equation (10) generates $z_{1:d}$ and affects only $y_{1:d}$. At the same time, the determinant of the Jacobian is tractable, which relies on the fact that:

$$\begin{array}{cc} \frac{\partial (f_{b}\circ f_{a})}{\partial \alpha_{a}^{T}}\left(\alpha_{a}\right) & =\frac{f_{a}}{\partial \alpha_{a}^{T}}\left(\alpha_{a}\right)\cdot \frac{f_{b}}{\partial \alpha_{b}^{T}}\left(\alpha_{b}=f_{a}\left(\alpha_{a}\right)\right),\\ \det(A\cdot B) & =\det(A)\cdot \det(B). \end{array}$$

The Jacobian matrices of these transformation functions are as follows:

$$\frac{\partial f_{\theta }}{\partial x}=\left[\begin{array}{cc} I_{1:d} & 0\\ \frac{\partial y_{d+1:D}}{\partial x_{1:d}^{T}} & \frac{\partial y_{d+1:D}}{\partial x_{d+1:D}^{T}} \end{array}\right],\frac{\partial f_{\theta }}{\partial y}=\left[\begin{array}{cc} \frac{\partial z_{1:d}}{\partial y_{1:d}^{T}} & \frac{\partial z_{d+1:D}}{\partial y_{d+1:D}^{T}}\\ 0 & I_{d+1:D} \end{array}\right].$$

It is noted that the Jacobian matrices of the transformation functions are upper triangular matrix and lower triangular matrix respectively, which simplify the calculation of the Jacobian determinants. In order to calculate the logarithmic probability of the transformation distribution, we need the help of inverse transformation functions and the inverse logarithmic Jacobian determinants. The logarithmic probability can be computed as follows.

(11) $$\pi_{u}\left(x\right)=q\left(f_{\theta }^{-1}\left(x\right)\right)\det\left|\frac{\partial f_{\theta }^{-1}}{\partial x}\right|,$$

where *q* represents the initial distribution. The inverse transformation functions $f_{\theta }^{-1}$ take the form as:

(12) $$\left\{\begin{array}{c} y_{d+1:D}=z_{d+1:D},\\ y_{1:d}\;\;\;=\left(z_{1:d}-T\left(y_{d+1:D}\right)\right)⊙\exp\left(-Sy_{d+1:D}\right))\\ \;\;\;\;-\epsilon \cdot \exp\left(\sigma \left(y_{d+1:D}\right)\right)+\frac{\epsilon^{2}}{2}\cdot \nabla U{\left(\left(t_{1},y_{d+1:D}\right)\right)}_{1:d},\\ t_{1}\;\;\;=\left(z_{1:d}-T\left(y_{d+1:D}\right)\right)⊙\exp\left(-S\left(y_{d+1:D}\right)\right)-\epsilon \cdot \exp(\sigma \left(y_{d+1:D}\right). \end{array}\right.$$

It is noted that Equation (12) is approximately invertible. Since we introduce gradient information to the transformation functions, the inverse transformation function $f_{\theta }^{-1}$ is difficult to obtain. For instance, in Equation (12), $z_{1:D}$ is known and we wish to use $z_{1:D}$ to calculate $y_{1:D}$. Although we can easily obtain $y_{d+1:D}$ through the first equation of Equation (12), when it comes to calculating $y_{1:d}$, we have to calculate $\nabla U{\left(y_{1:D}\right)}_{1:d}$ to update $y_{1:d}$. However achieving the closed-form solution for $y_{1:d}=\frac{\epsilon^{2}}{2}\cdot \nabla U{\left(y_{1:D}\right)}_{1:d}+const$ is difficult especially when the gradient function is complex, where $const=\left(z_{1:d}-T\left(y_{d+1:D}\right)\right)⊙\exp\left(-S\left(y_{d+1:D}\right)\right)-\epsilon \cdot \exp\left(\sigma \left(y_{d+1:D}\right)\right)$. In order to calculate $y_{1:d}$, we have additionally introduced a variable $t_{1}$ in the process of calculating the inverse transformation function. We set $y_{1:D}$ in $\nabla U{\left(y_{1:D}\right)}_{1:d}$ to be $\left(t_{1},y_{d+1:D}\right)$ and we calculate $t_{1}$ without using gradient information. Finally we update $y_{1:d}$ through $\nabla U{\left(\left(t_{1},y_{d+1:D}\right)\right)}_{1:d}$. The error of this approximation is $\frac{\epsilon^{2}}{2}\left[\nabla U{\left(y_{1:D}\right)}_{1:d}-\nabla U{\left(\left(t_{1},y_{d+1:D}\right)\right)}_{1:d}\right]$ which depends on the product of $\frac{\epsilon^{2}}{2}$ and $\nabla U\left(\left(\xi ,y_{d+1:D}\right)\right),\xi \in \left(y_{1:d},t_{1}\right)$. This approach is also exploited in the calculation of $x_{1:D}$, which takes the form as:

(13) $$\left\{\begin{array}{c} x_{1:d}\;\;\;=y_{1:d},\\ x_{d+1:D}=\left(y_{d+1:D}-T\left(x_{1:d}\right)\right)⊙\exp\left(-S\left(x_{1:d}\right)\right)\\ \;\;\;\;\;-\epsilon \cdot \exp\left(\sigma \left(x_{1:d}\right)\right)+\frac{\epsilon^{2}}{2}\cdot \nabla U{\left(\left(x_{1:d},t_{2}\right)\right)}_{d+1:D},\\ t_{2}\;\;\;=\left(y_{d+1:D}-T\left(x_{1:d}\right)\right)⊙\exp\left(-S\left(x_{1:d}\right)\right)-\epsilon \cdot \exp\left(\sigma \left(x_{1:d}\right)\right). \end{array}\right.$$

In order to calculate the logarithmic probability of the transformation distribution, we have to compute the inverse logarithmic Jacobian determinants. The final formulas are defined as follows:

(14) $$\begin{array}{cc} \ln\left|\frac{\partial f_{\theta }^{-1}}{\partial z}\right|= & \ln|\exp\left(-S\left(y_{d+1:D}\right)\right)+\frac{\epsilon^{2}}{2}\cdot \nabla \nabla U{\left(\left(t_{1},y_{d+1:D}\right)\right)}_{1:d}⊙\exp\left(-S\left(y_{d+1:D}\right)\right)|,\\ \ln\left|\frac{\partial f_{\theta }^{-1}}{\partial y}\right|= & \ln|\exp\left(-S\left(y_{1:d}\right)\right)+\frac{\epsilon^{2}}{2}\cdot \nabla \nabla U{\left(\left(x_{1:d},t_{2}\right)\right)}_{d+1:D}⊙\exp\left(-S\left(y_{1:d}\right)\right)|. \end{array}$$

Particularly, we introduce Langevin diffusions to normalization flows to construct the transformation function. Since the Langevin diffusions exploit the gradient of the target distribution, the transformation function is able to explore the state space efficiently.

Hamiltonian dynamics introduce the auxiliary momentum variable to explore the state space efficiently. Through the transformation of the energy over potential energy and kinetic energy, the total energy remains unchanged. Since the change of the state is associated with the transformation of the energy, designing normalization flows which are based on Hamiltonian dynamics becomes complex, which will be our future work.

### 3.2. Difference between Normalization Flows and Langevin Normalization Flows

There are two main differences between normalization flows and Langevin normalization flows. First, NFL cooperates with Langevin diffusions to construct an efficient and accurate approximation for the target distributions competing with the normalization flows. Second, when approximating the target distribution, the normalization flows are trained to minimize KL(q|p), where *q* represents the approximation distribution and *p* represents the target distribution. Since the transformation function is invertible, the integral of KL(q|p) can be calculated precisely. However, for NFL, the transformation functions demonstrated in Equations (12) and (13) are only approximately invertible because of the usage of the gradient information of the target distribution. Since the precise value of KL(q|p) cannot be obtained through integration, Monte Carlo estimation is used to calculate KL(q|p).

## 4. Dynamical Sampling Using Langevin Normalization Flows

Probabilistic inference involving multi-modal distributions is very difficult for dynamics based MCMC samplers. Besides, samples generated from these samplers are still highly auto-correlated. In order to solve these problems, we develop a new Monte Carlo sampler using Langevin normalization flows which are called Langevin normalization flows Monte Carlo (NFLMC). Given the target distribution and the initial distribution, NFLMC learns the parameters of the conversion of the initial distribution to the target distribution of the sampler. In the following subsections, we begin to describe the main idea of the method and then we introduce how our method works. Finally, we give the loss function of the training procedure and the algorithm. When the value of loss function converges, NFLMC can precisely sample from the target distribution.

### 4.1. Main Idea

The procedure of NFLMC is elaborated here. Assume that the target distribution is denoted as $\pi_{t}$, the initial distribution is denoted as $\pi_{q}$, θ represents the parameters of the transformation functions, $\pi_{u}$ represents the transformation distribution and Ls represents the Langevin step length. First, we generate *N* samples $X={\left\{x_{\left(t\right)}\right\}}_{t=0}^{N},x\in R^{D}$ from $\pi_{q}$ and initialize the parameters θ in the transformation functions. For each sample x∈X, the update equation takes the form as:

(15) $$\left\{\begin{array}{c} x_{1:d}\;\;\;=x_{1:d},\\ x_{d+1:D}=x_{d+1:D}-\frac{\epsilon^{2}}{2}\nabla U{\left(x_{1:D}\right)}_{d+1:D}+\epsilon \cdot \exp\left(\sigma \left(x_{1:d}\right)\right). \end{array}\right.$$

We repeatedly utilize Equation (15) Ls times to update $x_{d+1:D}$, where ϵ is the step size of Langevin diffusions, $\nabla U{\left(x_{1:D}\right)}_{d+1:D}$ is the gradient of the energy function of the target distribution. It is noted that the second term in Equation (15) is similar with Equation (8). After applying Ls steps of Langevin diffusions, we rescale $x_{d+1:D}$ through Equation (9) and we obtain $y_{1:D}$ which is a half update of $x_{1:D}$. We then update $x_{1:d}$ which takes the form as:

(16) $$\left\{\begin{array}{c} y_{d+1:D}=y_{d+1:D},\\ y_{1:d}\;\;\;=y_{1:d}-\frac{\epsilon^{2}}{2}\nabla U{\left(y_{1:D}\right)}_{1:d}+\epsilon \cdot \exp\left(\sigma \left(y_{d+1:D}\right)\right). \end{array}\right.$$

We also repeatedly utilize Equation (16) Ls times to update $x_{1:d}$. After that we rescale $x_{1:d}$ through Equation (10) and finally we obtain $z_{1:D}=f_{\theta }\left(x_{1:D}\right)$, where we define the transformation function as $f_{\theta }$. Now we gain the samples $z_{1:D},z_{1:D}∼\pi_{u}$. In order to optimize the parameters θ in $f_{\theta }$ to close to the target distribution. Through minimizing $\mathrm{KL}(\pi_{u}|\pi_{t})$, we are able to obtain the optimal parameters of $f_{\theta }$. Since the integral of $\mathrm{KL}(\pi_{u}|\pi_{t})$ for Langevin normalization flow is intractable, we use the Monte Carlo integral to calculate $\mathrm{KL}(\pi_{u}|\pi_{t})$. The objective function is as follows:

(17) $$\min_{\theta }\mathrm{KL}\left(\pi_{u}|\pi_{t}\right)=\frac{1}{N}\min_{\theta }\sum_{i=1,x_{\left(i\right)}∼\pi_{u}}^{N}\ln\frac{\pi_{u}\left(x_{\left(i\right)}\right)}{\pi_{t}\left(x_{\left(i\right)}\right)}.$$

As Equation (17) shows, we need the samples generated from $\pi_{u}$ and the probability of each sample to calculate the loss function. Since we have already had $z_{1:D}$ generated from $\pi_{u}$, we only need to calculate the logarithmic probability for $\pi_{u}\left(z_{1:D}\right)$ which takes the form as:

(18) $$\ln\left[\pi_{u}\left(z_{1:D}\right)\right]=\ln\left[\pi_{q}\left(f_{\theta }^{-1}\left(z_{1:D}\right)\right)\right]+\ln\left(\det\left|\frac{\partial f_{\theta }^{-1}}{\partial z_{1:D}}\right|\right),$$

where $f_{\theta }^{-1}$ is the inverse transformation function which can be calculated through Equation (12) and Equation (13). Since the update of $x_{1:D}$ is divided into two parts, the calculation of $\ln\left(\det\left|\frac{\partial f_{\theta }^{-1}}{\partial z_{1:D}}\right|\right)$ takes the form as:

(19) $$\ln\left(\det\left|\frac{\partial f_{\theta }^{-1}}{\partial z_{1:D}}\right|\right)=\ln\left(\det\left|\frac{\partial f_{\theta }^{-1}}{\partial z_{1:d}}\right|\right)+\ln\left(\det\left|\frac{\partial f_{\theta }^{-1}}{\partial z_{d+1:D}}\right|\right),$$

where $\ln\left(\det\left|\frac{\partial f_{\theta }^{-1}}{\partial z_{1:d}}\right|\right)$ and $\ln\left(\det\left|\frac{\partial f_{\theta }^{-1}}{\partial z_{d+1:D}}\right|\right)$ can be written as:

(20) $$\begin{array}{cc} \ln\left|\frac{\partial f_{\theta }^{-1}}{\partial z_{1:d}}\right|= & \ln|\exp\left(-S\left(z_{d+1:D}\right)\right)+\frac{\epsilon^{2}}{2}\cdot \nabla \nabla U{\left(\left(t_{1},z_{d+1:D}\right)\right)}_{1:d}⊙\exp\left(-S\left(z_{d+1:D}\right)\right)|,\\ \ln\left|\frac{\partial f_{\theta }^{-1}}{\partial z_{d+1:D}}\right|= & \ln|\exp\left(-S\left(z_{1:d}\right)\right)+\frac{\epsilon^{2}}{2}\cdot \nabla \nabla U{\left(\left(y_{1:d},t_{2}\right)\right)}_{d+1:D}⊙\exp\left(-S\left(y_{1:d}\right)\right)|, \end{array}$$

where $y_{1:d}$, $t_{1}$ and $t_{2}$ can be calculated through Equations (12) and (13).

However, in the progress of optimizing Equation (17), we find that the KL divergence may not be strictly non-negative because of the Monte Carlo integral, so we introduce a new objective function to overcome this problem. The detailed content is discussed in the next subsection.

### 4.2. Loss Function of the Training Procedure

As we have already discussed the transformation function in Langevin normalization flows, we do need a criterion to ensure that the final transformation distribution $\pi_{u}$ will converge to the target distribution $\pi_{t}$. In order to train the parameters θ which control the function σ, *S* and *T*, we choose to minimize $\mathrm{KL}(\pi_{u}|\pi_{t})$ as the loss function to guarantee that $\pi_{u}$ will be the expected distribution. Specifically, we take the advantage of Monte Carlo sampling to calculate the integral in KL divergence. Although the KL divergence is non-negative in theory, Monte Carlo integral may cause the abnormal of the result which means that the KL divergence is negative. In that case, minimizing Equation (17) will enable the loss to be smaller and thus the transformation distribution will not converge to the correct direction. To address this problem, we propose a new loss function which is defined as follows:

(21) $$\begin{array}{cc} L_{\pi_{u}\to \pi_{t}}\left(\theta \right) & =\int \pi_{u}\left(x\right){\left(\ln\frac{\pi_{u}\left(x\right)}{\pi_{t}\left(x\right)}\right)}^{2}dx=E_{\pi_{u}}\left[{\left(\ln\frac{\pi_{u}\left(x\right)}{\pi_{t}\left(x\right)}\right)}^{2}\right]. \end{array}$$

Since we have $E_{\pi_{u}}\left[{\left(\ln\frac{\pi_{u}\left(x\right)}{\pi_{t}\left(x\right)}\right)}^{2}\right]\geq E_{\pi_{u}}^{2}\left[\ln\frac{\pi_{u}\left(x\right)}{\pi_{t}\left(x\right)}\right]$, it is reasonable for us to minimize $E_{\pi_{u}}\left[{\left(\ln\frac{\pi_{u}\left(x\right)}{\pi_{t}\left(x\right)}\right)}^{2}\right]$ to achieve the purpose of minimizing the KL divergence.

### 4.3. Unnormalized Probability Distributions

In Bayesian machine learning, we generally require sampling from the posterior distribution to approximate the complex probabilistic modal. Since p(θ|D)∝p(D|θ)p(θ), the posterior distribution is an unnormalized distribution. So, we discuss the unnormalized probability distributions in this section. We assume that the unnormalized probability distribution $p_{unt}\left(x\right)$ equals to $\pi_{t}\left(x\right)Z$, where *Z* is the true normalization constant and $\pi_{t}$ is the probability density function. After utilizing the Equation (21), we observe that:

(22) $$\begin{array}{cc} L_{\pi_{u}\to p_{unt}}\left(\theta \right)= & \int \pi_{u}\left(x\right)\ln^{2}\frac{\pi_{u}\left(x\right)}{\pi_{t}\left(x\right)}dx+\ln^{2}Z\cdot \int \pi_{u}\left(x\right)dx\\ \; & +2\ln Z\cdot \int \pi_{u}\left(x\right)\ln\frac{\pi_{u}\left(x\right)}{\pi_{t}\left(x\right)}dx. \end{array}$$

It is noted that the third term $2\ln Z\cdot \int \pi_{u}\left(x\right)\ln\frac{\pi_{u}\left(x\right)}{\pi_{t}\left(x\right)}dx$ in Equation (22) can be simplified as:

(23) $$\begin{array}{c} 2\ln Z\cdot \int \pi_{u}\left(x\right)\ln\frac{\pi_{u}\left(x\right)}{\pi_{t}\left(x\right)}dx=2\ln Z\cdot \mathrm{KL}\left(\pi_{u}|\pi_{t}\right). \end{array}$$

The object of the optimization is to minimize the loss function $L_{\pi_{u}\to p_{unt}}$, which is equivalent to minimize $\int \pi_{u}\left(x\right)\ln^{2}\frac{\pi_{u}\left(x\right)}{\pi_{t}\left(x\right)}dx$ and $2\ln Z\cdot \mathrm{KL}(\pi_{u}|\pi_{t})$, for $\ln^{2}Z$ is a constant. Since the KL divergence is nonnegative, if Z∈(0,1), then $2\ln Z\cdot \mathrm{KL}(\pi_{u}|\pi_{t})$ is negative. Minimizing $2\ln Z\cdot \mathrm{KL}(\pi_{u}|\pi_{t})$ is to maximizing $\mathrm{KL}(\pi_{u}|\pi_{t})$, which will mislead the direction of the optimization.

So as to solve this problem, we introduce a scale parameter γ. We assume $p_{unt}\left(x\right)=\frac{\pi_{t}\left(x\right)Z}{\gamma }$, so the loss function can be written as:

(24) $$\begin{array}{cc} L_{\pi_{u}\to p_{unt}}\left(\theta \right)= & \int \pi_{u}\left(x\right)\ln^{2}\frac{\pi_{u}\left(x\right)}{\pi_{t}\left(x\right)}dx+\ln^{2}\frac{Z}{\gamma }\cdot \int \pi_{u}\left(x\right)dx+2\ln\frac{Z}{\gamma }\cdot \int \pi_{u}\left(x\right)\ln\frac{\pi_{u}\left(x\right)}{\pi_{t}\left(x\right)}dx\\ = & \frac{1}{N}\sum_{x_{\left(i\right)}\;∼\pi_{u}}^{N}\ln^{2}\frac{\pi_{u}\left(x_{\left(i\right)}\right)}{\pi_{t}\left(x_{\left(i\right)}\right)}+2\ln\frac{Z}{\gamma }\cdot \frac{1}{N}\sum_{x_{\left(i\right)}\;∼\pi_{u}}^{N}\ln\frac{\pi_{u}\left(x_{\left(i\right)}\right)}{\pi_{t}\left(x_{\left(i\right)}\right)}+\ln^{2}\frac{Z}{\gamma }\\ = & E_{\pi_{u}}\left[\ln^{2}\frac{\pi_{u}\left(x\right)}{\pi_{t}\left(x\right)}\right]+2\ln\frac{Z}{\gamma }\cdot E_{\pi_{u}\left(x\right)}\left[\ln\frac{\pi_{u}\left(x\right)}{\pi_{t}\left(x\right)}\right]+\ln^{2}\frac{Z}{\gamma }. \end{array}$$

As Equation (24) hinted, the function is composed of three terms. The first term is the same as Equation (21). The second term is the scaling term and the last term is a constant term. If γ=Z, then we recover the Equation (21). If γ<Z, then $2\ln\frac{Z}{\gamma }\cdot E_{\pi_{u}}\left[\ln\frac{\pi_{u}}{\pi_{t}}\right]$ is nonnegative, which not only ensures that the loss function will optimize towards the right direction but also cooperates with the information of KL divergence. In addition, the parameter γ is able to control the force of the optimization of KL divergence.

Furthermore, it is noted that the gradient of the loss function is:

(25) $$\begin{array}{cc} \nabla_{\theta }L_{\pi_{u}\to p_{unt}}\left(\theta \right)= & 2\sum_{i=1,x_{\left(i\right)}\;∼\pi_{u}}^{N}\ln\frac{\pi_{u}\left(x_{\left(i\right)}\right)}{\pi_{t}\left(x_{\left(i\right)}\right)}\nabla_{\theta }\left[\ln\frac{\pi_{u}\left(x_{\left(i\right)}\right)}{\pi_{t}\left(x_{\left(i\right)}\right)}\right]+2\ln\frac{Z}{\gamma }\cdot \sum_{i=1,x_{\left(i\right)}\;∼\pi_{u}}^{N}\nabla_{\theta }\left[\ln\frac{\pi_{u}\left(x_{\left(i\right)}\right)}{\pi_{t}\left(x_{\left(i\right)}\right)}\right], \end{array}$$

where $2\sum_{i=1,x_{\left(i\right)}\;∼\pi_{u}}^{N}\ln\frac{\pi_{u}\left(x_{\left(i\right)}\right)}{\pi_{t}\left(x_{\left(i\right)}\right)}\nabla_{\theta }\left[\ln\frac{\pi_{u}\left(x_{\left(i\right)}\right)}{\pi_{t}\left(x_{\left(i\right)}\right)}\right]$ gives the importance weight $\ln\frac{\pi_{u}\left(x_{\left(i\right)}\right)}{\pi_{t}\left(x_{\left(i\right)}\right)}$ for $\nabla_{\theta }\left[\ln\frac{\pi_{u}\left(x_{\left(i\right)}\right)}{\pi_{t}\left(x_{\left(i\right)}\right)}\right]$, for each sample $x_{\left(i\right)}$, so it can be viewed as the rescale of the gradient of the KL divergence, which proves the correctness of the loss function. The complete algorithm is given in Algorithm 1.

**Algorithm 1** Training NFLMC**Input:**  target distribution $\pi_{t}$, step size ϵ, learning rate β, scale parameter γ, Langevin step length Ls, number of iterations $K_{iters}$, sample number *N*, the initial distribution $\pi_{q}$, the transformation distribution $\pi_{u}$, the energy function *U*, the gradient of energy function ∇U and the second order gradient ∇∇U. **Output:**   the parameters $\theta =(W_{\sigma },W_{S},W_{T})$ of the sampler.    Initializing the parameters θ of the neural network.    **for**
k=1
**to**
$K_{iters}$
**do**        Sample *N* samples from the proposal distribution $\pi_{q}$.        $x∼\pi_{q},X={\left\{x_{\left(n\right)}\right\}}_{n=1}^{N}$        **for**
i=1
**to**
Ls
**do**            $x_{d+1:D}=x_{d+1:D}-\frac{\epsilon^{2}}{2}\nabla U{\left(x_{1:D}\right)}_{d+1:D}+\epsilon \cdot \exp\left(\sigma \left(x_{1:d}\right)\right)$
        Obtaining $y_{1:D}$ through Equation (9).        **for**
i=1
**to**
Ls
**do**            $x_{1:d}=y_{1:d}-\frac{\epsilon^{2}}{2}\nabla U{\left(y_{1:D}\right)}_{1:d}+\epsilon \cdot \exp\left(\sigma \left(y_{d+1:D}\right)\right)$        Obtaining $z_{1:D}$ through Equation (10).        Calculating the loss $L_{\pi_{u}\to p_{unt}}\left(\theta \right)$ through Equation (24).        Obtaining $\ln\pi_{u}$ by using Equation (18).        $L_{\pi_{u}\to p_{unt}}\left(\theta \right)=\frac{1}{N}\sum_{n=1}^{N}{\left(\ln\frac{\gamma \pi_{u}\left(z_{\left(n\right)}\right)}{p_{unt}\left(z_{\left(n\right)}\right)}\right)}^{2}$        Calculating $\nabla_{\theta }L_{\pi_{u}\to p_{unt}}\left(\theta \right)$ through Equation (25).        Updating θ in the transformation functions.        $\theta =\theta -\beta \nabla_{\theta }L_{\pi_{u}\to p_{unt}}\left(\theta \right)$

In practice, there are several important points to note about the implementation of Algorithm 1. First, the proposal distribution $\pi_{q}$ should be a simple distribution which is easy to analyze. We suggest to use the Gaussian distribution as the proposal distribution. Second, the number of the samples *N* should be set to a large value. In our experiments, we set N=8000. Third, the scale parameter γ can be estimated through importance sampling. $\gamma =\sum_{x∼q(x)}\frac{Z\pi_{t}}{q(x)}$, where $Z\pi_{t}$ represents the target distribution and q(x) represents the proposal distribution. We have built a demo program which is available at: https://github.com/Emcc81/NFLMC.

## 5. Applicability of NFLMC

In this section, we will demonstrate the performance of NFLMC. We present a detailed analysis of our trained sampler on varieties of target distributions. First, we will compare the proposed sampler with RNVP and HMC on five different distributions which are composed of the ring (the ring-shaped density), the ill-conditioned Gaussian, the strongly correlated Gaussian, the Gaussian funnel and the rough Well. After that, we present the results on two multi-modal distributions. Finally, we demonstrate the results on a task from machine learning —Bayesian logistic regression.

All our experiments are conducted on a standard computer with eight Nvidia RTX2080Ti GPUs. The nodes of each layer of the neural networks are set to be 512 with ReLU as the activation function. The number of the layer of the neural networks is set to be 3. Langevin steps are set to be 2 to 5. The number of transformation functions is set to be 8. The learning rate is set to be 0.05. The maximum iteration is set to be 10,000. We estimate scale parameter γ through importance sampling. Now, we introduce the performance index which will be used in the following parts.

Effective sample size—The variance of a Monte Carlo sampler is determined by its effective sample size (ESS) [14] which is defined as:

(26) $$\mathrm{ESS}=N/(1+2\times \sum_{s=1}^{M}\rho \left(s\right)),$$

where *N* represents the total sampling number, *M* is set to be 30 in our experiments and ρ(s) represents the *s*-step autocorrelation. Autocorrelation is an index which considers the correlation between two samples. Let *X* be a set of samples and *t* be the number of iteration (*t* is an integer). Then $X_{t}$ is the sample at time *t* of *X*. The definition of the autocorrelation between time *s* and *t* is:

(27) $$R\left(s,t\right)=\frac{E[\left(X_{t}-\mu_{t}\right)\left(X_{s}-\mu_{s}\right)]}{\sigma_{t}\sigma_{s}},$$

where E is the expected value operator. Autocorrelation can measure the correlation between two nearby samples. If the value of autocorrelation is high, the samples are far from independent and vice versa.

Maximum mean discrepancy—The difference between samples drawn from two distributions can be measured as maximum mean discrepancy (MMD) [43] which is defined as follows:

(28) $$\begin{array}{cc} MMD^{2}\left[X,Y\right]= & \frac{1}{M^{2}}\sum_{i,j=1}^{M}k\left(x_{i},x_{j}\right)-\frac{2}{MN}\sum_{i,j=1}^{M,N}k\left(x_{i},y_{j}\right)+\frac{1}{N^{2}}\sum_{i,j=1}^{N}k\left(y_{i},y_{j}\right), \end{array}$$

where *M* represents the sample number in *X*, *N* represents the sample number in *Y* and *k* represents the kernel function. Through MMD, we can analyze the convergence speed of the proposed methods.

### 5.1. Varieties of Unimodal Distributions

Since RNVP performs well in density estimation, we utilize the loss function proposed in Equation (21) to train RNVP to sample from the target distribution. This kind of method is called the naive normalization flows Monte Carlo (NNFMC). We then compare the NFLMC with NNFMC and HMC on convergence rate and autocorrelation, respectively. In each experiment, we set the same learning rate for NFLMC and NNFMC. The initial distributions are all set to be the standard normal distribution. We next introduce the distributions used in the experiment.

Ring: The ring shaped target density. The analytic form of the energy function of the ring is: $U\left(x\right)=\frac{{\left(\sqrt{x_{1}^{2}+x_{2}^{2}}-2\right)}^{2}}{0.32}$.

Ill-conditioned Gaussian: Gaussian distribution with diagonal covariance spaced log-linearly between $10^{-2}$ and $10^{2}$.

Strongly correlated Gaussian: We rotate a diagonal Gaussian with variances $\left[10^{2},10^{-2}\right]$ by $\frac{\pi }{4}$. This is an extreme version of an example from Brooks [14].

Rough well: A similar example from Sohl-Dickstein et al. [44] and its energy function is: $U\left(x\right)=\frac{1}{2}x^{T}x+\eta \sum_{i}\cos\left(\frac{x_{i}}{\eta }\right)$. We set $\eta =10^{-2}$.

Gaussian funnel: We conduct our experiment on a 2-*D* funnel, whose energy function takes the form as: $U\left(x\right)=\frac{1}{2}\left[{\left(\frac{x_{1}}{\sigma }\right)}^{2}+\frac{x_{2}^{2}}{\exp(x_{1})}+\ln\left(2\pi \cdot \exp(x_{1})\right)\right]$ and we set σ=1.0.

**Figure 2:**
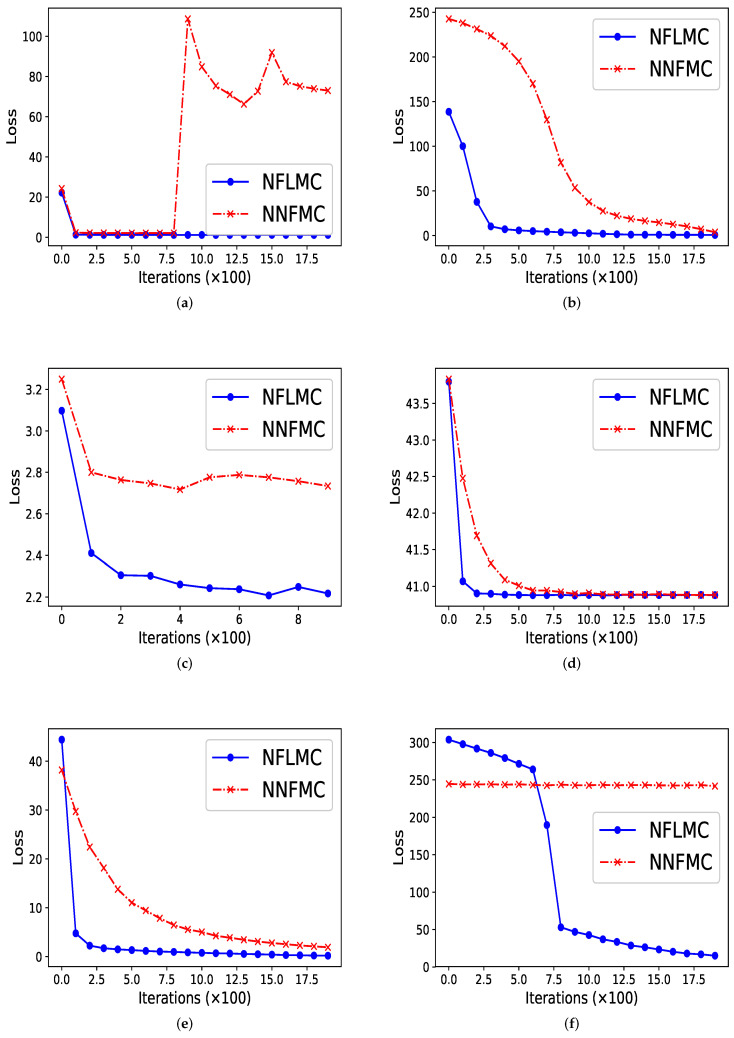
The comparison of normalization flows Monte Carlo (NFLMC) and naive normalization flows Monte Carlo sampler (NNFMC) on six different distributions. The abscissa represents the number of iterations and the ordinate represents the value of loss in training procedure. (**a**) The performance of NFLMC and NNFMC on ring. (**b**) The performance of NFLMC and NNFMC on ill conditioned Gaussian. (**c**) The performance of NFLMC and NNFMC on rough well. (**d**) The performance of NFLMC and NNFMC on Gaussian funnel. (**e**) The performance of NFLMC and NNFMC on strongly correlated Gaussian with μ=[0,0]. (**f**) The performance of NFLMC and NNFMC on strongly correlated Gaussian with μ=[10,10].

As Figure 2 illustrates, our method performs better in all these distributions in terms of convergence rate. In ill conditioned Gaussian, rough well, Gaussian funnel and strongly corrected Gaussian with μ=[0,0], NFLMC gains fast convergence, which indicates that the Langevin diffusions do help the normalization flows to find the correct direction. In strongly corrected Gaussian with μ=[10,10], NNFMC is unable to converge to the target distribution, since the loss remains high during the training procedure. It is the utilization of gradient information of the target distribution that aids NFLMC to converge the target distribution rapidly.

**Figure 3:**
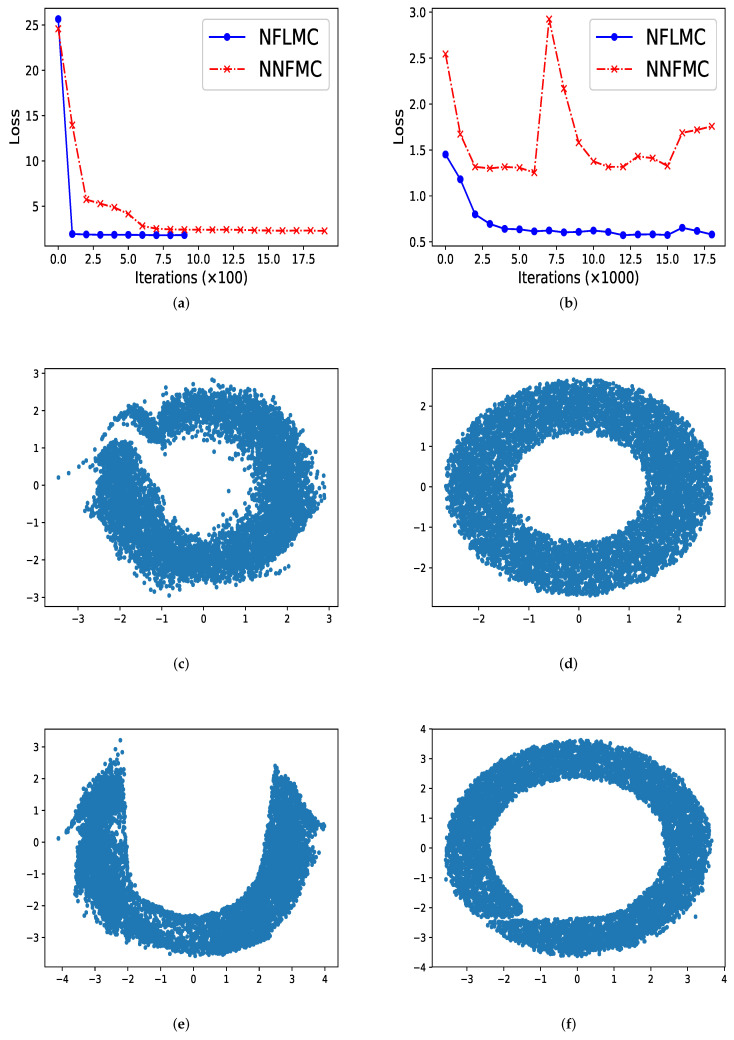
The performance of naive normalization flows Monte Carlo (NNFMC) and Langevin normalization flows Monte Carlo (NFLMC) on two ring distributions. Specially, for the ring distributions with large radius, we show the change of the loss with respect to iterations after 1000 iterations. (**a**) The loss of NFLMC and NNFMC on a ring with small radius. (**b**) The loss of NFLMC and NNFMC on a ring with large radius. (**c**) The scatter diagram of NNFMC on a ring with small radius. (**d**) The scatter diagram of NFLMC on a ring with small radius. (**e**) The scatter diagram of NNLMC on a ring with large radius. (**f**) The scatter diagram of NFLMC on a ring with large radius.

In ring-shaped distribution, NNFMC has a significant fluctuation in the process of training, while NFLMC converges rapidly during the training procedure, which shows the stability of NFLMC. Since the loss of NNFMC has large fluctuation, we carefully tune the learning rate for NNFMC. As Figure 3 illustrates, NFLMC converges to the target distribution more quickly than NNFMC. Besides, NNFMC has a large error while NFLMC is able to sample from the target distribution precisely.

So what causes the large fluctuation of NNFMC? We think that the lack of strong guidance when exploring the state space makes NNFMC difficult to converge. Since the initial distribution is a standard normal distribution, samples from the initial distribution have large distance with the samples of ring-shaped distribution. So it is challenging for NNFMC to explore state space and the value of the loss function has large fluctuation. In order to verify this thought, we enlarge the size of the ring distribution whose energy function has the form: $U\left(x\right)=\frac{{\left(\sqrt{x_{1}^{2}+x_{2}^{2}}-3\right)}^{2}}{0.32}$ and we observe that NNFMC fails to sample from this distribution while NFLMC can still converge to the target distribution. As Figure 3 shows, NNFMC cannot find the target distribution, while NFLMC still performs well, for NFLMC utilizes the gradient information of the target distribution.

We then compare our method with HMC in terms of the autocorrelation on five different distributions.

**Figure 4:**
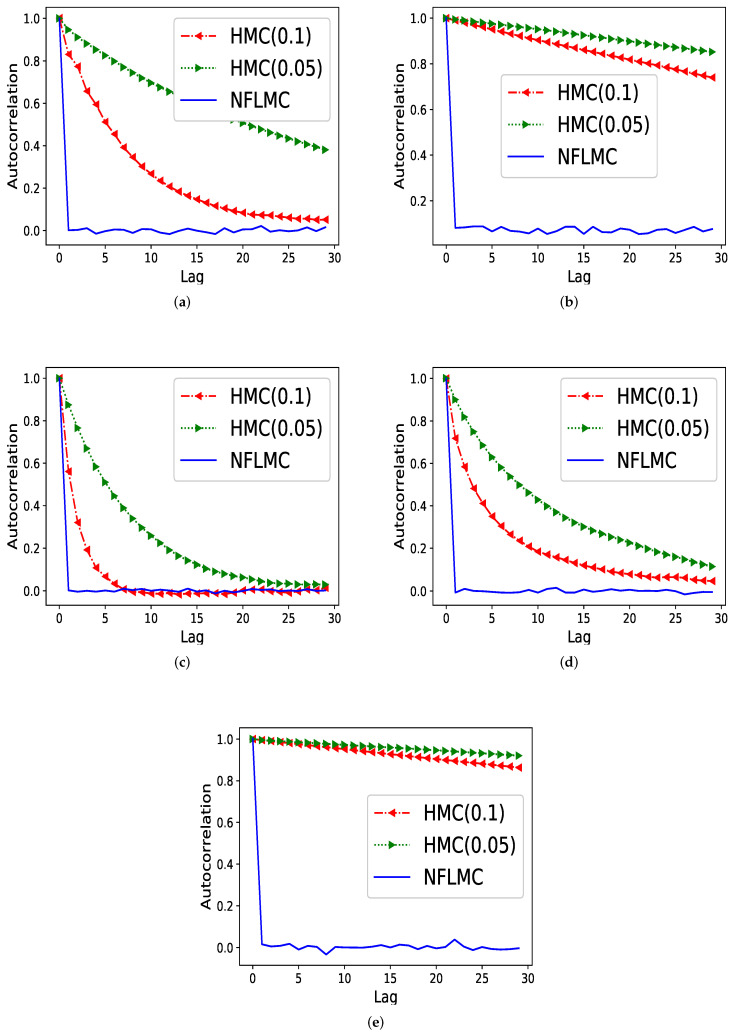
The comparison of Hamiltonian Monte Carlo (HMC) and NFLMC on the autocorrelation on five different distributions. The abscissa represents the steps between the samples and the ordinate represents the autocorrelation between samples. (**a**) The autocorrelation of HMC and NFLMC on ring. (**b**) The autocorrelation of HMC and NFLMC on ill conditioned Gaussian. (**c**) The autocorrelation of HMC and NFLMC on rough well. (**d**) The autocorrelation of HMC and NFLMC on Gaussian funnel. (**e**) The autocorrelation of HMC and NFLMC on strongly correlated Gaussian with μ=[0,0].

Figure 4 demonstrates that NFLMC obtains better performance in autocorrelation, which indicates that NFLMC overcomes the defects of the MCMC samplers. HMC (0.05) and HMC (0.1) represent the HMC sampler with different step size.

### 5.2. Mixtures of Gaussian Distributions

We conduct our second experiment on two multi-modal distributions where we consider two simple 2-*D* mixtures of Gaussian distributions (MOG) whose probability density function are analytically available. First, we consider a MOG whose modes have the same probability and then we consider a MOG whose modes have different probabilities and further distance. The first distribution is defined as: $p\left(x\right)=\frac{1}{2}N\left(x|\mu ,I\right)+\frac{1}{2}N\left(x|-\mu ,I\right)$, where μ=(2.5,−2.5). The second distribution is defined as: p(x)=0.88N(x|μ,I)+0.12N(x|−μ,I), where μ=(4,−4). The experiment settings is the same with Tripuraneni et al. [24]. The purpose of the experiments is to sample points which are i.i.d. distributed in multi-modal distributions correctly.

**Figure 5:**
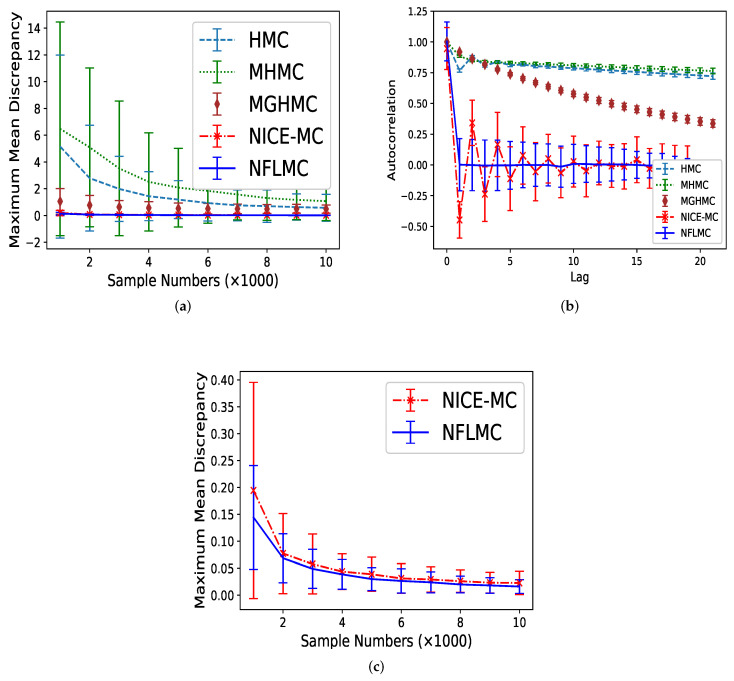
The comparison of five different methods on maximum mean discrepancy (MMD) and autocorrelation on Gaussian mixtures distribution. (**a**) The relationship between autocorrelation and sample numbers. (**b**) The relationship between autocorrelation and lag. (**c**) The detailed comparison of NFLMC and NICE-MC on MMD.

We compare HMC [14], MHMC [24], MGHMC [30] and NICE-MC [29] against NFLMC. First, we compare the MMD of these methods and then averaged autocorrelation is used to compare the performance of each method further. Each MCMC method is run 32 times and 20,000 iterations with 11,000 burn-in samples. The number of leap-frog steps is uniformly drawn from (100−l,100+l) with l=20, which is suggested by Livingstone et al. [45]. We set step size ϵ=0.05 and the initiate position x=(0,0). The initial distribution for NFLMC is a Gaussian distribution with μ=[0,0] and diag(σ)=[2,2]. As Figure 5 illustrates, NFLMC obtains excellent performance compared with MHMC, HMC and MGHMC regarding MMD and autocorrelation. In addition, NFLMC has a smaller variance of MMD compared with NICE-MC. However, when it comes to autocorrelation, NICE-MC shows the huge fluctuation, while NFLMC remains steady, which manifests the stability of NFLMC.

**Figure 6:**
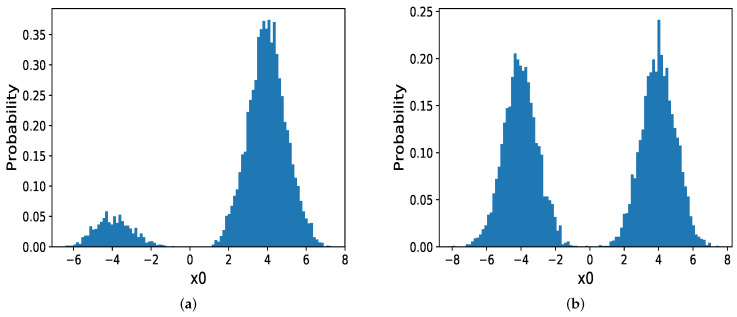
The performance of parallel HMC and NFLMC on the mixtures of Gaussian with different probabilities of the modes. (**a**) The histogram of NFLMC on $x_{0}$. (**b**) The histogram of parallel HMC on $x_{0}$.

We then discuss the circumstance in which the modes are far from each other and with different probabilities. When μ in MOG become larger, for instance, μ=(4,−4). In Hamiltonian dynamics, there exists a significant force in this low probability regions which hinder samplers from jumping out of the current mode. In other words, the gradients in boundary regions are tremendous and the momentum will increasingly decrease until it changes its direction which makes HMC and MHMC challenging to sample from the target distribution. So we compare NFLMC with parallel HMC and NICE-MC. The scatter diagram of both parallel HMC and NFLMC is demonstrated in Figure 6. We observe that parallel HMC can sample from the multi-modal distribution but it cannot precisely estimate the probability of each mode.

**Figure 7:**
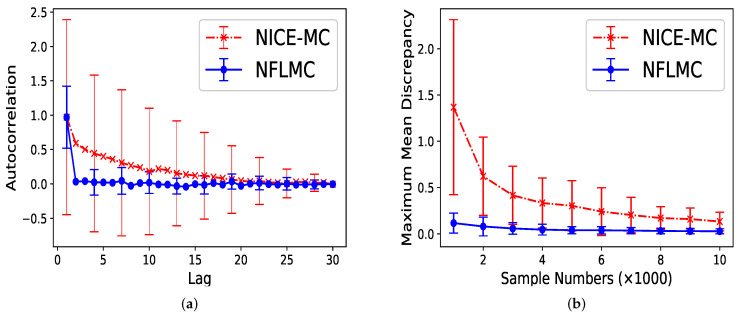
The performance of NFLMC and NICE-MC on the mixtures of Gaussians with different probabilities of the modes. (**a**) The autocorrelation of the samples generated from NFLMC and NICE-MC. (**b**) The change of MMD on NFLMC and NICE-MC.

For parallel HMC, it seems that two modes have the same probability. However, the real probability of each mode is $\pi_{1}=0.12,\pi_{2}=0.88$. As Figure 7 illustrates, compared with NICE-MC, NFLMC converges quickly to the target distribution while gains the lower autocorrelation. It is the fact that NFLMC takes advantage of the neural networks to explore the phase space, which results in good performance.

### 5.3. Bayesian Logistic Regression

Logistic regression (LR) [46] is a traditional way for classification. Employing maximizing the logistic likelihood function, we can get the optimized parameters. Through the parameters, we can predict the class of the data. Bayesian logistic regression [47] is also a classic model for classification which takes advantage of logistic sigmoid function as the likelihood function. For the two-class classification, the likelihood function is defined as: $p\left(t|w\right)=\prod_{n=1}^{N}{\left[1-y_{n}\right]}^{1-t_{n}}$, where $t={\left(t_{1},\ldots ,t_{N}\right)}^{⊤}$ and $y_{n}=p\left(C_{1}|\phi_{n}\right)=\sigma \left(w^{⊤}\phi \right)$. $t_{n}$ represents the category of the data and $y_{n}$ represents the probability of the data belonging to one class. Through integrating the logistic function on the posterior distribution, we can get the class of the data. However, sometimes the integral is difficult to calculate, variational Bayesian logistic regression (VBLR) substitute the real posterior distribution to the variational distribution. Instead of using variational inference, we apply Monte Carlo sampling technology to this model. Through sampling from the posterior distribution, the class of the data can be estimated.

We evaluate our methods on nine real-world datasets from UCI repository [48]—Pima Indian (Pi), Haberman (Ha), Blood (Bl), Immunotherapy (Im), Indian (In), Mammographic (Ma), Heart (He), German (Ge) and Australian (Au) using Bayesian logistic regression. Feature dimensions are from 3 to 25 and the data instances are from 306 to 1086. All datasets are normalized to have zero mean value and unit variance. First, we set the standard normal distribution N(0,I) as the prior distribution for the parameters. In each experiment, we run 9000 iterations with 1000 burn-in samples for HMC. For NFLMC, we set the standard normal distribution as the initial distribution. We train the NFLMC sampler until the value of loss function converges and then sampling 8000 samples using the well-trained sampler. The maximum number of iterations is set to be $10^{5}$. We set the step size ϵ=0.001 and we run ten times to calculate the mean and the standard deviation.

Results regarding the accurate rate of prediction and area under the receiver operating characteristic curve (AUC) [49] are summarized in Table 1 and Table 2, respectively. The results show that in these nine datasets, NFLMC yields good performance in accurate rate and AUC. In order to further compare the quality of the samples, we calculate the mean of ESS of each dimension for both HMC and NFLMC. We use 30 steps autocorrelation to calculate this value. Table 3 demonstrates that NFLMC achieves higher ESS than HMC, which suggests that NFLMC has lower autocorrelation than HMC for each dimension.

## 6. Discussion and Conclusions

In this study, we propose Langevin normalization flows and develop Langevin normalization flows Monte Carlo, a novel scalable sampling algorithm which exploits the flexibility of the neural networks and efficient exploration of Langevin diffusions. We design the appropriate loss function to train the sampler to ensure that the sampler is able to converge to the target distribution. We also discuss the unnormalized probability distributions and propose the appropriate loss function to these distributions. The experiments conducted on synthetic and real datasets suggest that our method is able to sample from the target distributions precisely and independently.

Although HMC has various advantages, it is difficult for us to design the model based on HMC, because the auxiliary momentum variable should be carefully concerned in the transformation function of NFs. In the future, we plan to design the neural network sampler based on Hamiltonian dynamics.

## Figures and Tables

**Table 1:** Classification accuracy for variational Bayesian logistic regression (VBLR), logistic regression (LR), HMC and NFLMC on nine different datasets.

Data	LR	VBLR	HMC	NFLMC
Ha	69.3 ± 0.2	69.3 ± 0.1	69.3 ± 0.2	69.4 ± 0.1
Pi	76.6 ± 0.2	76.2 ± 0.1	76.6 ± 0.1	76.6± 0.1
Ma	82.5 ± 0.3	83.1 ± 0.1	83.1 ± 0.1	83.1 ± 0.2
Bl	76.0 ± 0.2	76.0 ± 0.2	76.0 ± 0.3	76.0 ± 0.1
Im	77.7 ± 0.3	77.75 ± 0.4	83.2 ± 0.2	83.3 ± 0.2
In	75.8 ± 0.3	73.2 ± 0.2	73.2 ± 0.2	74.1 ± 0.2
He	75.9 ± 0.2	75.9 ± 0.2	75.9 ± 0.2	75.9 ± 0.1
Ge	71.5 ± 0.1	71.5 ± 0.1	72.5 ± 0.2	73.0 ± 0.1
Au	86.9 ± 0.2	87.6 ± 0.2	87.6 ± 0.2	87.7 ± 0.1

**Table 2:** Area under the receiver operating characteristic curve (AUC) for VBLR, LR, HMC and NFLMC on nine different datasets.

Data	LR	VBLR	HMC	NFLMC
Ha	62.7 ± 0.1	63.2 ± 0.1	63.0 ± 0.2	63.2 ± 0.1
Pi	79.2 ± 0.2	79.3 ± 0.1	79.3 ± 0.1	79.5 ± 0.1
Ma	89.9 ± 0.1	89.8 ± 0.1	89.89 ± 0.1	89.9 ± 0.2
Bl	73.5 ± 0.3	73.4 ± 0.3	74.4 ± 0.3	73.5 ± 0.2
Im	76.7 ± 0.3	78.5 ± 0.5	89.2 ± 0.2	89.3 ± 0.3
In	73.2 ± 0.3	73.2 ± 0.2	72.4 ± 0.2	72.8 ± 0.4
He	80.1 ± 0.2	81.3 ± 0.2	82.2 ± 0.3	84.8 ± 0.2
Ge	74.7 ± 0.2	75.5 ± 0.2	76.7 ± 0.3	76.9 ± 0.1
Au	92.5 ± 0.2	93.9 ± 0.2	93.9 ± 0.3	94.0 ± 0.2

**Table 3:** The mean of effective sample size of each dimension for HMC and NFLMC on nine differen datasets.

Data	HMC	NFLMC	Data	HMC	NFLMC
Ha	107.69	2503.75	In	408.87	3590.34
Pi	73.08	3534.50	He	1093.10	3200.00
Ma	670.72	2570.69	Ge	7.19	2842.92
Bl	808.84	2824.87	Au	220.60	2538.25
Im	1879.78	1917.54
